# Supplementary material for: Expanding and improving analyses of nucleotide recoding RNA-seq experiments with the EZbakR suite
Source: PLoS Comput Biol. 2025 Jul 3;21(7):e1013179. doi: 10.1371/journal.pcbi.1013179 (PMC12251217; doi:10.1371/journal.pcbi.1013179)
Supplement: Attachment — Submitted filename: EZbakR_rebuttal.docx [file pcbi.1013179.s003.docx]

We thank the reviewers for their insightful comments and suggestions, all of which we address in this revised manuscript. The revised manuscript includes the following two major additions and changes:

1. We have added several new analyses of real datasets to showcase and validate the novel functionalities introduced in EZbakR. This complements the analyses of real data included in our original submission (impact of hierarchical mutation rate modeling in Figure 4, exploration of processing kinetics in Figure S3, and identification of PUNDs in Figure S6, now S7) and includes:
   a. A reanalysis of a previously published TimeLapse-seq dataset (Courvan et al. 2024) in which multiple perturbations are made in parallel, and that benefits from EZbakR’s generalized linear modeling. The original authors used bakR and had to rerun bakR multiple times to make all the comparisons they were interested in. EZbakR significantly streamlines this process and allows all such comparisons to be made with a single model fit (Figure 6F and S8).
   b. Comparative analyses of subcellular dynamics using data from Ietswaart et al. 2024. EZbakR identifies the impacts of DDX3X knockdown on synthesis, nuclear export, and cytoplasmic degradation kinetics. This analysis corroborates what Ietswaart et al. found, that DDX3X’s primary direct effect seems to be on nuclear export kinetics. This highlights a powerful and exciting use case of EZbakR, which is to kinetically dissect the impact of biological perturbations with unprecedented resolution.
2. We have added comparisons of EZbakR with existing tools in the cases where existing tools implement some form of an analysis strategy discussed. This includes:
   a. Comparisons of EZbakR’s modeling of pre-mRNA dynamics (Figure 5A) to INSPEcT (Figure S3A and B). INSPEcT is only able to fit this one model, whereas EZbakR generalizes dynamical systems modeling of NR-seq data. In addition, INPSPEcT is not optimized for NR-seq analyses, and thus requires EZbakR to provide fraction new estimates as input for accurate kinetic parameter estimates. We show that using a naïve mutational cutoff for fraction new estimation, instead of EZbakR’s output from mixture modeling output, significantly biases INSPEcT’s kinetic parameter estimates (Figure S3B).
   b. Comparisons of EZbakR’s differential kinetic analysis with grandR (Figure 6B-E). We show that grandR is orders of magnitude less efficient than EZbakR, requiring hours to analyze a simulated dataset that EZbakR analyzes in under a minute, and is significantly underpowered relative to EZbakR.

Our revisions are complemented by our recent work (Mabin and Vock et al. 2025, *bioRxiv*) that includes an additional suite of simulated and real data validations of EZbakR and further demonstrates the usefulness of generalized feature assignment for the quantification of isoform-specific kinetics and the identification of transcripts targeted by NMD.

We hope the reviewers agree that the revised manuscript has significantly improved and addresses all the comments from their reviews. We reiterate our gratefulness for the time and effort the reviewers put into carefully assessing our work. Below is a point-by-point response.

**Reviewer #1**

**In this manuscript, the authors present a new version of their software bakR, called EZbakR, for performing differential kinetics analysis on RNA metabolism.
I have no significant concerns regarding the methods or the implementation, the online documentation and the supplementary methods are relatively complete. However, I believe that the manuscript itself is premature and lacks in scholarly presentation, and most results presented are simulated,except for Figures 4C and related supplementary figures, unless I'm mistaken, or consist in schematics, without context or adequate description, sometimes requiring an effort to understand how the figures relate to the text.**

We thank the reviewer for their kind words regarding our detailed method sections and documentation, as well as their suggestions for improvements of this manuscript. We have edited several figures and associated text to increase clarity and scholarship and have added several new analyses of real data. More specifically, as discussed above and further below, we have complemented real data analyses from our original submission (those in 4C referenced by the reviewer, as well as analyses of pre-mRNA processing kinetics in Figure S3 and identification of instances of robust nuclear degradation in Figure S6 (S7 in the revised text)) with two new analyses to showcase the usefulness of EZbakR’s generalized linear modeling (Figure 6F) and the unique potential to assess changes in subcellular RNA kinetics by combining EZbakR’s generalized linear dynamical systems modeling with its efficient and well powered comparative analyses (Figure 7, reproduced below).

Figure 7: EZbakR identifies the effects of DDX3X knockdown on subcellular RNA kinetics. **A)** Model to which nuclear, cytoplasmic, and total RNA NR-seq data from Ietswaart et al. was fit. **B)** EZbakR comparative analyses of synthesis, export, and degradation kinetics with and without DDX3X knockdown. **C)** Changes in synthesis, export, and degradation kinetics for DDX3X target and non-target transcripts. Red dotted lines represent median changes in each kinetic parameter for the non-targets.

**In "generalized and improved mixture modeling", an example of feature-specific incorporation rate is shown using MT, but what other "features" can be used, if any use-cases is known, and how would this approach generalize?**

We thank the reviewer for this question. We have edited the wording in that section and in the figure legend to clarify what is being shown. The feature-specific incorporation rate is estimated for each feature, which in this case is a gene (exonic regions only). Thus, each gene gets its own *p*_labeled_ estimate. In Figure 4C, we are coloring points by whether that gene is encoded on the mitochondrial chromosome, as recent work has suggested that RNA transcribed from these genes specifically have lower incorporation rates. Our gene-specific incorporation rate estimates corroborate this finding and would generalize to any setting where RNA transcribed from different genes may have different label incorporation rates.

**For "generalized linear dynamical systems modeling" and "generalized comparative analyses", no real data are shown. It is difficult to evaluate the capabilities of EZbakR. Uses cases of modeling flow between subcellular compartments is particularly interesting, but it's not clear how much of
data from 14 was used for this. I don't see any significant results for "generalized comparative analyses", and nothing regarding experimental perturbations, etc. For this particular aspect, and in general for visualization, I don't see how EZbakR is better than grandR.**

**Overall, I believe well-designed uses-cases should be presented to validate the new features proposed in this work, either by using published data with sufficient detail and relevance, either by producing new data, or at least for simulated data, by providing a comparative analysis with established tools that may in some cases provide similar analyses such as grandR, or e.g. for modeling of premature and mature RNA expression, INSPEcT, etc.**

We thank the reviewer for these suggestions and have added 1) comparisons of EZbakR to INSPEcT for the task of assessing pre-mRNA synthesis, processing, and mature RNA degradation kinetics 2) comparisons of EZbakR to grandR on simulated data 3) an example of how generalized linear modeling streamlines analyses of a previously published dataset originally analyzed with bakR, and 4) a demonstration of how generalized dynamical systems modeling combined with EZbakR’s comparative analyses allows it to pinpoint the gene-specific set of subcellular kinetic impacts of DDX3X knockdown.

1) We show that when INSPEcT is provided with labeled and unlabeled RNA abundances derived from EZbakR’s mixture models, its estimates of synthesis, processing, and turnover kinetics are of similar accuracy to those from EZbakR. INSPEcT is not optimized for NR-seq analyses and does not implement a mixture model for analyzing NR-seq data. We thus also assessed INSPEcT’s performance on simulated data when provided with new and old RNA abundances from a simple mutation cutoff (1 or more mutation = new), and show that its kinetic parameter estimates in this case are often highly biased (Figure S3A and B).
2) We show that comparative analyses of kinetics using grandR is not only orders of magnitude less efficient than with EZbakR (taking hours vs. tens of seconds to analyze the simulated dataset), but that grandR by all metrics is significantly underpowered relative to EZbakR (Figure 6B-E). grandR’s function for this use case (EstimateRegulation()) provides a credible interval of a user-specified width for each parameter estimate, as well as a region of practical equivalence (ROPE) probability that the true L2FC lies within a user-specified range (-0.25 to 0.25 by default). The former is directly comparable to EZbakR’s output, and is termed the grandR (CI) method in Figure 6, but the latter is not. We thus devised a strategy to optimize a cutoff for the ROPE probability that yielded FDR control on-par with that of the highest power model (bakR MCMC), and found that even in this unreasonably fine-tuned case, grandR is still significantly underpowered relative to EZbakR (Figure 6D; grandR (oracle) method).
3) The greatest strength of design matrix specified linear modeling in EZbakR is that it facilitates performing comparative analyses in more complex experimental settings. For example, if two perturbations are being applied, there are often a number of different comparisons that users will want to make, and/or interaction effects they would like to assess. This was the case in a recent publication using bakR (Courvan et al. 2024). For that work, the authors analyzed NR-seq data from various combinations of activated/resting and normoxic/hypoxic macrophages. Doing this with bakR required multiple reruns of bakR, one for each set of comparisons to a given reference condition. EZbakR allows users to make all desired comparisons with a single model fit, thus greatly streamlining this analysis (Figure 6F and S8).
4) A unique and exciting use-case of EZbakR is that it combines well powered, efficient comparative analyses with flexible kinetic parameter estimation. This allowed us to compare subcellular RNA flow kinetics across biological conditions. Ietswaart et al. previously combined subcellular NR-seq with DDX3X knockdown. They were able to identify global changes in nuclear export kinetics, but they lacked a strategy to identify specific genes that were high confidence instances of this change. EZbakR’s reanalysis of this data confirms the nuclear export defect, identifies a number of high confidence export affected genes, and shows strong correlation between this set and DDX3X targets identified by a previous eCLIP dataset. This showcases the power of EZbakR to dissect the set of kinetic impacts of biological perturbations (Figure 7).

The real data analyses added complement the real data analyses included in our original submission (analyses of pre-mRNA processing and degradation kinetics in K562 cells (Figure S3) and identification of PUNDs using the Ietswaart et al. subcellular NR-seq data (originally Figure S6, now Figure S7)).

**In fact for most results presented in "generalized linear dynamical systems modeling", "fraction labeled mixing model" was used, this is the ideal case if no spike-ins are available. But anyhow a comparison should be made with "fraction labeled modeling" and, ideally, with spike-ins.**

We thank the reviewer for their suggestion and have added a comparison of the accuracy of spike-in normalization with the fraction labeled mixing model on simulated data (Figure S4). It shows that fraction labeled mixing provides comparably accurate parameter estimates. No published subcellular NR-seq data has included a full suite of spike-ins capable of providing normalization across all samples/compartments. We thus are unable to demonstrate the effectiveness of this normalization strategy on real data and have implemented it in EZbakR only as a forward-looking option in the case such an experimental normalization strategy is eventually developed.

**I also have a number of rather more pedantic comments, but I don't understand why the authors are trying to redefine an already established terminology. The authors coined the term "nucleotide recoding RNA sequencing methods or NR-seq" in a number of their own publications, e.g. 10.1093/nar/gkac693, 10.1261/rna.079451.122 or preprints such as 10.1101/2023.05.24.542133, etc. however this terminology leads to confusion, it is not generally used, and could be falsely understood to refer to the (biological) concept of recoding. The authors themselves describe NR-seq as experiments involving "chemically converting s4U from a uridine analog to a cytidine analog". So why not stick to common usage e.g. "nucleotide conversion", "4sU-tagging", or "s6G-tagging", etc. ?**

We thank the reviewer for the opportunity to clarify our chosen naming convention and have added a reference to the term “nucleotide conversion RNA-seq” in our introduction to increase clarity. When we first proposed the term NR-seq, no standard term existed. Everyone would just refer to the specific chemistry used (e.g. SLAM-seq) or use the more general term “metabolic labeling” that does not capture the specifics of these methods. 4sU-tagging is often used to specifically refer to enrichment-based methods that involve conjugating biotin to 4sU-containing RNA, not enrichment free approaches. We wanted a chemistry agnostic term, and went with nucleotide recoding RNA-seq as we thought it more accurately reflected all existing chemistries. SLAM chemistry does not convert the hydrogen bonding pattern of s^4^U to C (it disrupts it in a way that gets it reverse transcribed to a G), and the typical amine used for TimeLapse chemistry yields a cytosine analog rather than fully converting it to a native C. Thus, we felt that it was more accurate to state that these chemistries were recoding s^4^U to look like a C to reverse transcriptases. Most users of these methods continue to refer to the particular chemistry used, and nucleotide conversion RNA-seq has really only been widely adopted in papers from the Erhard lab (GRAND-SLAM and grandR developers) and their collaborators, who originally proposed it in the grandR preprint, released shortly after the bakR preprint. Thus, given our existing widespread use of the term NR-seq (i.e., in the work mentioned by the reviewer as well as all our extensive documentation for bakR, bam2bakR, EZbakR, and fastq2EZbakR) and the lack of a fully definitive chemistry agnostic alternative, we have respectfully decided to mostly stick with our use of the term “NR-seq” in the revised manuscript.

**Besides, when they refer to NR-seq, in the context of this manuscript, i.e. for kinetics analysis/modelling, kinetic parameter estimation, they essentially refer to TimeLapse-seq, SLAM-seq, and TUC-seq, or their derivatives. These three methods are so far the most used metabolic labelling methods for these types of analysis. So why NR-seq? Why adding a "etc." to this list?**

We appreciate the reviewer’s perspective and question. EZbakR was specifically designed to be broadly compatible with NR-seq methods. In addition to the distinct chemistries used in TimeLapse-seq, SLAM-seq, and TUC-seq, other chemistries have been developed since these three, and while we agree none of these have yet seen widespread adoption, we include “etc.” when listing the set of NR-seq methods to respect those contributions and acknowledge the possibility that additional NR-seq methods will become widely used. This emphasis is reflected in the concluding sentence of our manuscript, “We anticipate that the EZbakR sutie will provide a unified bioinformatic platform to support the ever-expanding NR-seq ecosystem.”

**Finally, a rather large paragraph of the introduction is dedicated to various methods (what they refer to as NR-seq) using s4U metabolic labeling in one way or another, but not necessarily within the context of kinetic modelling as referred to in this manuscript, e.g. references 8, 10, or 17 (authors cite their own work). Some references are even not relevant, i.e. not using 4sU-tagging at all, at least as far as I know, e.g. 11 and 13. I don't see why this is necessary, this just misleads the reader.**

We thank the reviewer for their comments and have reworked that entire paragraph to increase clarity. Not all these citations are meant to be of NR-seq methods, and the EZbakR suite is not only meant to support analyses of kinetics. We included citations to Ribo-seq, PacBio long read sequencing, a single-cell RNA-seq review, and Start-seq when referencing the NR-seq extension of these methods. In addition, not all NR-seq methods are designed to provide kinetic parameter estimates. EZbakR’s generalized feature assignment and mixture modeling are still applicable in these settings. We thus respectfully disagree with the reviewer and have kept all citations, while clarifying these points in the text.

**In the introduction, the authors mention pulseR and SLAMDUNK, and say that kinetic parameter estimation is "dependent on the metabolic label incorporation rate". They mention their own proposed method for TimeLapse-seq, which eventually is similar to the GRAND-SLAM approach. However, their mixture model approach, that of pulseR, or GRAND-SLAM, all rely on some "mutational analysis", as they refer to, which ultimately boils down to some form of identification of labeled vs. unlabeled. pulseR and GRAND-SLAM were previously compared across different labelling methods, the authors should check this paper**

We thank the reviewer for their comments and have clarified the text to better reflect the argument we are trying to make here. Our point, made in Figure S1, is that strategies like pulseR and SLAMDUNK that do not implement rigorous mixture modeling are subject to biases that are a function of metabolic label incorporation efficiency. These tools rely on simple mutational cutoffs to distinguish reads from labeled and unlabeled RNAs. A particular cutoff will only provide unbiased fraction labeled estimates in a narrow range of incorporation rates, as we showed in Figure S1. Outside of this range, they will become systematically biased. That is what we mean when we refer to these methods being “dependent on the metabolic label incorporation rate”. In the paper that the reviewer has mentioned, an attempt is made to account for this bias via a modification to pulseR’s standard model, though the authors note that “for the chemical conversion [methods, pulseR] shows more variability (more parameters to fit)”, further highlighting the optimality of mixture modeling.

**The authors also say that "grandR [does not] support well-powered comparative analyses akin to those implemented in differential expression analysis software". I must say that I have not used grandR, but looking at the documentation, I tend to disagree with the authors. grandR appears to be much more flexible than GRAND-SLAM, allows to access data and metadata, including intermediate data, and perform various DE analyses. In all cases, the authors fail to provide a fair comparison between EZbakR and grandR.**

We thank the reviewer for their comments and have walked back this statement, but we point to our analyses mentioned above (Figure 6 B-E) that confirm that grandR significantly underperforms EZbakR on the task of performing comparative analyses. In addition, when we criticize GRAND-SLAM for not providing intermediate mutational data, we mean data that goes into a mixture model. grandR takes the output of GRAND-SLAM (or theoretically other mixture models, though this is more cumbersome) as input, and thus does nothing to overcome this key limitation.

**Reviewer #2**

**The authors developed an R package, EZbakR, and a corresponding Snakemake pipeline to expand the possible analyses for nucleotide recoding sequencing data such as NR-seq, TimeLapse-seq, SLAM-seq, TUC-seq, and others. The tool successfully estimates simulated parameters based on differential equations models derived from steady state kinetics. Following parameter estimation, the package can also perform generalized linear regression inference on any estimated parameter to identify possible changes between conditions. Overall, this software will be a valuable addition to facilitate the analysis of several existing and new datasets that aim to record the kinetics of RNA metabolism. Moreover, the authors have done an excellent job maintaining the codebase, ensuring it is well-structured and sufficiently documented for execution.**

We thank the reviewer for their support of our work and their kind words regarding our efforts to maintain and document our codebases.

**The authors mostly use simulations to highlight accuracy. This is acceptable given the lack of a gold standard. They also provide some real data applications of the tool to showcase qualitatively meaningful results. Since the authors have used the data from Ietswaart et al., 2024, it is important to also provide comparisons to the methodology used in that publication. Currently only a small subset of this comparison is presented in Figure S6. This analysis needs to be expanded so the readers can evaluate expected differences between the two workflows.**

We thank the reviewer for their suggestions and have added a more direct comparison to kinetic parameter estimates from the original Ietswaart et al. study (Figure S6, reproduced below). This comparison shows that EZbakR largely recapitulates kinetic parameter estimates in the K562 data from that paper. EZbakR also reveals the practical identifiability challenges of estimating polysome loading kinetics, explaining the particularly strong deviation between kinetic parameter estimates in this case (Figure S6C and D). Similarly to the identifiability issues discussed in Figure 5D, this is likely due to polysome loading being highly efficient, and by extension the cytoplasmic RNA being largely polysome bound and thus providing limited information about the loading kinetics. A more systematic investigation of differences in the Ietswaart et al. workflow is made difficult as, per correspondence with the authors, the original pipeline takes over a month to run on their datasets (vs. less than 10 hours from fastqs to kinetic parameter estimates for the EZbakR suite). It is also not implemented in a documented software package and is rather a collection of specialized scripts, and they plan to use EZbakR for analyses of all new subcellular TimeLapse-seq datasets that they generate.

Figure S6: EZbakR corroborates previously reported subcellular kinetics. **A)** Full subcellular flow model described in Ietswaart et al. (1). CH = chromatin bound RNA; NP = nucleoplasmic RNA; CY = cytoplasmic RNA; PL = polysome bound RNA. **B)** Comparison of kinetic parameter estimates for those with absolute quantitative units and consistently low EZbakR quantified uncertainty (< 0.3). Red dotted line represents perfect agreement. Points are colored by density. **C)** Comparison of previous polysome loading rate constant estimates to estimates from EZbakR. A much higher uncertainty cutoff of 1 is used here. See **D** for motivation. **D)** Comparison of the distributions of estimate uncertainties for all parameters. Violin plots overlay scatter plots of the full distribution of uncertainties with random horizontal jitter. Consistently high uncertainties of the polysome loading rate constant (k_pl_) are likely due to the efficiency of this step relative to cytoplasmic degradation. Thus, most RNA in the cytoplasmic fraction comes from polysome bound rather than free RNA, making it difficult to estimate the rate at which the latter becomes the former.

We also refer the reviewer to first set of points made in the summary at the top of this review as well as to reviewer 1 discussing the set of additional analyses of real datasets in this revised manuscript, including comparative analyses of subcellular kinetics upon DDX3X knockdown, which corroborate another set of general conclusions and trends presented in the Ietswaart et al study.

**Recently another tool, Nanodynamo, was published (PMID: 39231948, Nat. Comm) to perform similar analysis for long-read direct RNA sequencing with nanopores. While Nanodynamo has a different starting point (i.e. long reads), once the labeled and unlabeled reads are identified, one could use Nanodynamo to infer kinetic rates similar to EZbakR. Given that more tools are appearing to support NR-seq with direct RNA sequencing (PMID: 39211330) it would be beneficial for the authors to discuss EZbakR applicability to such datasets and potentially compare to Nanodynamo.**

We thank the reviewer for the suggestions and have added a sentence in the Discussion about EZbakR’s ability to make use of output from these specialized pipelines, as well as a figure comparing EZbakR’s estimates to those in Tarrero et al. … Pelizzola, PMID 39231948 (Figure S9). We find good concordance between the two approaches. While direct RNA sequencing differs from canonical NR-seq and is beyond the scope of fastq2EZbakR, EZbakR’s flexibility allows it to accept as input estimates of labeled and unlabeled RNA abundances. Thus, EZbakR can fit the models discussed in Tarrero et al. using the output of the pipeline discussed there. The reverse is not true, as Nanodynamo has a small subset of specialized hardcoded models it is capable of fitting. We also note that Nanodynamo appears to be at a much earlier stage of development (it is currently a collection of scripts to reproduce the exact analyses presented in that paper) and is not a fully distributable software package. For example, there is not yet documentation describing how to generalize beyond the paper-specific analyses, and the scripts have limitations, such as certain analyses only being able to accept data with a single label time.
